# Correctness of Sequential Monte Carlo Inference for Probabilistic Programming Languages

**DOI:** 10.1007/978-3-030-72019-3_15

**Published:** 2021-03-23

**Authors:** Daniel Lundén, Johannes Borgström, David Broman

**Affiliations:** Imperial College, London, UK; 9 Digital Futures and EECS, KTH Royal Institute of Technology, Stockholm, Sweden; 10 Uppsala University, Uppsala, Sweden

**Keywords:** Probabilistic Programming, Sequential Monte Carlo, Operational Semantics, Functional Programming, Measure Theory

## Abstract

Probabilistic programming is an approach to reasoning under uncertainty by encoding inference problems as programs. In order to solve these inference problems, probabilistic programming languages (PPLs) employ different inference algorithms, such as sequential Monte Carlo (SMC), Markov chain Monte Carlo (MCMC), or variational methods. Existing research on such algorithms mainly concerns their implementation and efficiency, rather than the correctness of the algorithms themselves when applied in the context of expressive PPLs. To remedy this, we give a correctness proof for SMC methods in the context of an expressive PPL calculus, representative of popular PPLs such as WebPPL, Anglican, and Birch. Previous work have studied correctness of MCMC using an operational semantics, and correctness of SMC and MCMC in a denotational setting without term recursion. However, for SMC inference—one of the most commonly used algorithms in PPLs as of today—no formal correctness proof exists in an operational setting. In particular, an open question is if the resample locations in a probabilistic program affects the correctness of SMC. We solve this fundamental problem, and make four novel contributions: (i) we extend an untyped PPL lambda calculus and operational semantics to include explicit resample terms, expressing synchronization points in SMC inference; (ii) we prove, for the first time, that subject to mild restrictions, any placement of the explicit resample terms is valid for a generic form of SMC inference; (iii) as a result of (ii), our calculus benefits from classic results from the SMC literature: a law of large numbers and an unbiased estimate of the model evidence; and (iv) we formalize the bootstrap particle filter for the calculus and discuss how our results can be further extended to other SMC algorithms.

* This project is financially supported by the Swedish Foundation for Strategic Research (ASSEMBLE RIT15-0012) and the Swedish Research Council (grant 2013-4853).

## 1 Introduction

Probabilistic programming is a programming paradigm for probabilistic models, encompassing a wide range of programming languages, libraries, and platforms [5,13,14,25,32,37,38]. Such probabilistic models are typically created to express *inference problems*, which are ubiquitous and highly significant in, for instance, machine learning [1], artificial intelligence [31], phylogenetics [29,30], and topic modeling [2].

In order to solve such inference problems, an *inference algorithm* is required. Common general-purpose algorithm choices for inference problems include *sequential Monte Carlo (SMC)* methods [9], *Markov chain Monte Carlo (MCMC)* methods [12], and *variational* methods [42]. In traditional settings, correctness results for such algorithms often come in the form of laws of large numbers, central limit theorems, or optimality arguments. However, for general-purpose probabilistic programming languages (PPLs), the emphasis has predominantly been on algorithm implementations and their efficiency [14,25,37], rather than the correctness of the algorithms themselves. In particular, explicit connections between traditional theoretical SMC results and PPL semantics have been limited. In this paper, we bridge this gap by formally connecting fundamental SMC results to the context of an expressive PPL calculus.

Essentially, SMC works by simulating many executions of a probabilistic program concurrently, occasionally *resampling* the different executions. In this resampling step, SMC discards less likely executions, and replicates more likely executions, while remembering the average likelihood at each resampling step in order to estimate the overall likelihood. In expressive PPLs, there is freedom in choosing where in a program this resampling occurs. For example, most SMC implementations, such as WebPPL [14], Anglican [43], and Birch [25], always resample when all executions have reached a call to the *weighting* construct in the language. At possible resampling locations, Anglican takes a conservative approach by dynamically checking during runtime if all executions have either stopped at a weighting construct, or all have finished. If none of these two cases apply, report a runtime error. In contrast, WebPPL does not perform any checks and simply includes the executions that have finished in the resampling step. There are also heuristic approaches [21] that automatically *align* resampling locations in programs, ensuring that all executions finish after encountering the same number of them. The motivations for using the above approaches are all based on experimental validation. As such, an open research problem is whether there are any inherent restrictions when selecting resampling locations, or if the correctness of SMC is independent of this selection. This is not only important theoretically to guarantee the correctness of inference results, but also for inference performance, both since inference performance is affected by the locations of resampling locations [21] and since dynamic checks result in direct runtime overhead. We address this research problem in this paper.

In the following, we give an overview of the paper and our contributions. In Section 2, we begin by giving a motivating example from phylogenetics, illustrating the usefulness of our results. Next, in Section 3, we define the syntax and operational semantics of an expressive functional PPL calculus based on the operational formalization in Borgström et al. [3], representative of common PPLs. The operational semantics assign to each pair of term $\mathbf{t}$ and initial random *trace* (sequences of random samples) a non-negative weight. This weight is accumulated during evaluation through a `weight` construct, which, in current calculi and implementations of SMC, is (implicitly) always followed by a resampling. To decouple resampling from weighting, we present our first contribution.

(i) We extend the calculus from Borgström et al. [3] to include explicit `resample` terms, expressing explicit synchronization points for performing resampling in SMC. With this extension, we also define a semantics which limits the number of evaluated resample terms, laying the foundation for the remaining contributions.

In Section 4, we define the probabilistic semantics of the calculus. The weight from the operational semantics is used to define unnormalized distributions $\langle\!\langle \mathbf{t} \rangle\!\rangle$ over traces and $[\![\mathbf{t}]\!]$ over result terms. The measure $[\![\mathbf{t}]\!]$ is called the *target measure*, and finding a representation of this is the main objective of inference algorithms.

We give a formal definition of SMC inference based on Chopin [6] in Section 5. This includes both a generic SMC algorithm, and two standard correctness results from the SMC literature: a law of large numbers [6], and the unbiasedness of the likelihood estimate [26].

In Section 6, we proceed to present the main contributions.

(ii) From the SMC formulation by Chopin [6], we formalize a sequence of distributions $\langle\!\langle \mathbf{t} \rangle\!\rangle_n$, indexed by $n$, such that $\langle\!\langle \mathbf{t} \rangle\!\rangle_n$ allows for evaluating at most $n$ `resample`s. This sequence is determined by the placement of `resample`s in $\mathbf{t}$. Our first result is Theorem 1, showing that $\langle\!\langle \mathbf{t} \rangle\!\rangle_n$ eventually equals $\langle\!\langle \mathbf{t} \rangle\!\rangle$ if the number of calls to `resample` is upper bounded. Because of the explicit `resample` construct, this also implies that, for *all* `resample` placements such that the number of calls to `resample` is upper bounded, $\langle\!\langle \mathbf{t} \rangle\!\rangle_n$ eventually equals $\langle\!\langle \mathbf{t} \rangle\!\rangle$. We further relax the finite upper bound restriction and investigate under which conditions $\lim_{n\to\infty} \langle\!\langle \mathbf{t} \rangle\!\rangle_n = \langle\!\langle \mathbf{t} \rangle\!\rangle$ pointwise. In particular, we relate this equality to the dominated convergence theorem in Theorem 2, which states that the limit converges as long as there exists a function dominating the weights encountered during evaluation. This gives an alternative set of conditions under which $\langle\!\langle \mathbf{t} \rangle\!\rangle_n$ converges to $\langle\!\langle \mathbf{t} \rangle\!\rangle$ (now asymptotically, in the number of resamplings $n$).

The contribution is fundamental, in that it provides us with a sequence of approximating distributions $\langle\!\langle \mathbf{t} \rangle\!\rangle_n$ of $\langle\!\langle \mathbf{t} \rangle\!\rangle$ that can be targeted by the SMC algorithm of Section 5. As a consequence, we can extend the standard correctness results of that section to our calculus. This is our next contribution.

(iii) Given a suitable sequence of transition kernels (ways of moving between the $\langle\!\langle \mathbf{t} \rangle\!\rangle_n$), we can *correctly* approximate $\langle\!\langle \mathbf{t} \rangle\!\rangle_n$ with the SMC algorithm from Section 5. The approximation is correct in the sense of Section 5: the law of large numbers and the unbiasedness of the likelihood estimate holds. As a consequence of (ii), SMC also correctly approximates $\langle\langle \mathbf{t} \rangle\rangle$, and in turn the target measure $[\![\mathbf{t}]\!]$. Crucially, this also means estimating the model evidence (likelihood), which allows for compositionality [15] and comparisons between different models [30]. This contribution is summarized in Theorem 3.

Related to the above contributions, Ścibior et al. [33] formalizes SMC and MCMC inference as transformations over monadic inference representations using a denotational approach (in contrast to our operational approach). They prove that their SMC transformations preserve the measure of the initial representation of the program (i.e., the target measure). Furthermore, their formalization is based on a simply-typed lambda calculus with primitive recursion, while our formalization is based on an untyped lambda calculus which naturally supports full term recursion. Our approach is also rather more elementary, only requiring basic measure theory compared to the relatively heavy mathematics (category theory and synthetic measure theory) used by them. Regarding generalizability, their approach is both general and compositional in the different inference transformations, while we abstract over parts of the SMC algorithm. This allows us, in particular, to relate directly to standard SMC correctness results.

Section 7 concerns the instantiation of the transition kernels from (iii), and also discusses other SMC algorithms. Our last contribution is the following.

(iv) We define a sequence of sub-probability kernels $k_{\mathbf{t},n}$ induced by a given program $\mathbf{t}$, corresponding to the fundamental SMC algorithm known as the *bootstrap particle filter (BPF)* for our calculus. This is the most common version of SMC, and we present a concrete SMC algorithm corresponding to these kernels. We also discuss other SMC algorithms and their relation to our formalization: the resample-move [11], alive [19], and auxiliary [28] particle filters.

Importantly, by combining the above contributions, we justify that the implementation strategies of the BPFs in WebPPL, Anglican, and Birch are indeed correct. In fact, our results show that the strategy in Anglican, in which every evaluation path must resample the same number of times, is too conservative.

An extended version of this paper is also available [20]. This extended version includes rigorous definitions and detailed proofs for many lemmas found in the paper, as well as further examples and comments. The lemmas proved in the extended version are explicitly marked with $^\dagger$.

### 2 A Motivating Example from Phylogenetics

In this section, we give a motivating example from phylogenetics. The example is written in a functional PPL[3] developed as part of this paper, in order to verify

[3] The implementation is an interpreter written in OCaml. It largely follows the same approach as Anglican and WebPPL, and uses continuation-passing style in order to

```
let tree = {
 left:{left:{age:0},right:{age:0},age:4},
 right:{left:{age:0},right:{age:0},age:6},
 age:10
} in let lambda = 0.2 in let mu = 0.1 in let crbdGoesExtinct startTime =
 let curTime = startTime
   - (sample (exponential (lambda + mu)))
 in
 if curTime < 0 then false
 else
  let speciation = sample
    (bernoulli (lambda / (lambda + mu))) in
  if !speciation then true
  else crbdGoesExtinct curTime
    && crbdGoesExtinct curTime in let simBranch startTime stopTime =
 let curTime = startTime -
   sample (exponential lambda) in
 if curTime < stopTime then ()
 else if not (crbdGoesExtinct curTime)
 then weight (log 0) // #1
 else (weight (log 2); // #2
       simBranch curTime stopTime) in let simTree tree parent =
 let w = -mu * (parent.age - tree.age) in
 weight w; // #3
 simBranch parent.age tree.age;
 match tree with
 | {left,right,age} ->
   simTree left tree; simTree right tree
 | {age} -> () in simTree tree.left tree;
simTree tree.right tree
```

Fig. 1: A simplified version of a phylogenetic birth-death model from [30]. See the text for a description.

and experiment with the presented concepts and results. In particular, this PPL supports SMC inference (Algorithm 2) with decoupled `resample`s and `weight`s[4], as well as sampling from random distributions with a `sample` construct.

Consider the program in Fig. 1, encoding a simplified version of a phylogenetic birth-death model (see Ronquist et al. [30] for the full version). The problem is to find the model evidence for a particular birth rate (`lambda = 0.2`) and death rate (`mu = 0.1`), given an observed phylogenetic `tree`. The tree represents known lineages of evolution, where the leaves are extant (surviving to the present) species. Most importantly, for illustrating the usefulness of the results in this paper, the recursive function `simBranch`, with its two `weight` applications `#1` and `#2`, is called a random number of times for each branch in the observed `tree`. Thus, different SMC executions encounter differing numbers of calls to `weight`. When resampling is performed after every call to `weight` (`#1`, `#2`, and `#3`), it is, because of the differing numbers of resamples, not obvious that inference is correct (e.g., the equivalent program in Anglican gives a runtime error). Our results show that such a resampling strategy is indeed correct.

This strategy is far from optimal, however. For instance, only resampling at `#3`, which is encountered the same number of times in each execution, performs much better [21,30]. Our results show that this is correct as well, and that it gives the same asymptotic results as the naive strategy in the previous paragraph.

Another strategy is to resample only at `#1` and `#3`, again causing executions to encounter differing numbers of resamples. Because `#1` weights with (log) 0, this pause and resume executions as part of inference. It is available at https://github.com/miking-lang/miking-dppl/tree/pplcore. The example in Fig. 1 can be found under `examples/crbd/crbd-esop.ppl`

[4] The implementation uses log weights as arguments to `weight` for numerical reasons.

approach gives the same accuracy as resampling only at `#3`, but avoids useless computation since a zero-weight execution can never obtain non-zero weight. Equivalently to resampling at `#1`, zero-weight executions can also be identified and stopped automatically at runtime. This gives a direct performance gain, and both are correct by our results. We compared the three strategies above for SMC inference with 50 000 particles[5]: resampling at `#1`,`#2`, and `#3` resulted in a runtime of 15.0 seconds, at `#3` in a runtime of 12.6 seconds, and at `#1` and `#3` in a runtime of 11.2 seconds. Furthermore, resampling at `#1`,`#2`, and `#3` resulted in significantly worse accuracy compared to the other two strategies [21,30].

Summarizing the above, the results in this paper ensure correctness when exploring different resampling placement strategies. As just demonstrated, this is useful, because resampling strategies can have a large impact on SMC accuracy and performance.

### 3 A Calculus for Probabilistic Programming Languages

In this section, we define the calculus used throughout the paper. In Section 3.1, we begin by defining the syntax, and demonstrate how a simple probability distribution can be encoded using it. In Section 3.2, we define the semantics and demonstrate it on the previously encoded probability distribution. This semantics is used in Section 4 to define the *target measure* for any given program. In Section 3.3, we extend the semantics of Section 3.2 to limit the number of allowed resamples in an evaluation. This extended semantics forms the foundation for formalizing SMC in Sections 6 and 7.

#### 3.1 Syntax

The main difference between the calculus presented in this section and the standard untyped lambda calculus is the addition of real numbers, functions operating on real numbers, a sampling construct for drawing random values from real-valued probability distributions, and a construct for weighting executions. The rationale for making these additions is that, in addition to discrete probability distributions, continuous distributions are ubiquitous in most real-world models, and the weighting construct is essential for encoding inference problems. In order to define the calculus, we let $X$ be a countable set of variable names; $D \in \mathbb{D}$ range over a countable set $\mathbb{D}$ of identifiers for families of probability distributions over $\mathbb{R}$, where the family for each identifier $D$ has a fixed number of real parameters $|D|$; and $g \in \mathbb{G}$ range over a countable set $\mathbb{G}$ of identifiers for real-valued functions with respective arities $|g|$. More precisely, for each $g$, there is a measurable function $\sigma_g : \mathbb{R}^{|g|} \to \mathbb{R}$. For simplicity, we often use $g$ to denote both the identifier and its measurable function. We can now give an inductive definition of the abstract syntax, consisting of values $\mathbf{v}$ and terms $\mathbf{t}$.

[5] We repeated each experiment 20 times on a machine running Ubuntu 20.04 with an Intel i5-2500K CPU (4 cores) and 8GB memory. The standard deviation was under 0.1 seconds in all three cases.

$\texttt{sample}_{Beta}(2,2)$

(a)

```
let p = sample_Beta(2, 2) in
let observe o =
  weight(f_Bern(p, o)) in
iter observe [true, false, true]; p
```

(b)

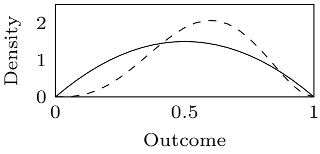

(c)

Fig. 2: The *Beta*(2, 2) distribution as a program in (a), and visualized with a solid line in (c). Also, the program $\mathbf{t}_{obs}$ in (b), visualized with a dashed line in (c). The *iter* function in (b) simply maps the given function over the given list and returns (). That is, it calls *observe true*, *observe false*, and *observe true* purely for the side-effect of weighting.

**Definition 1.**

$$\mathbf{v} ::= c \mid \lambda x.\mathbf{t} \qquad \begin{aligned} \mathbf{t} ::= \; & \mathbf{v} \mid x \mid \mathbf{t}\;\mathbf{t} \mid \texttt{if}\;\mathbf{t}\;\texttt{then}\;\mathbf{t}\;\texttt{else}\;\mathbf{t} \mid g(\mathbf{t}_1,\ldots,\mathbf{t}_{|g|}) \\ & \mid \texttt{sample}_D(\mathbf{t}_1,\ldots,\mathbf{t}_{|D|}) \mid \texttt{weight}(\mathbf{t}) \mid \texttt{resample} \end{aligned} \tag{1}$$

*Here, $c \in \mathbb{R}$, $x \in X$, $D \in \mathbb{D}$, $g \in \mathbb{G}$. We denote the set of all terms by $\mathbb{T}$ and the set of all values by $\mathbb{V}$.*

The formal semantics is given in Section 3.2. Here, we instead give an informal description of the various language constructs.

Some examples of distribution identifiers are $\mathcal{N} \in \mathbb{D}$, the identifier for the family of normal distributions, and $\mathcal{U} \in \mathbb{D}$, the identifier for the family of continuous uniform distributions. The semantics of the term $\texttt{sample}_{\mathcal{N}}(0,1)$ is, informally, "draw a random sample from the normal distribution with mean 0 and variance 1". The `weight` construct is illustrated later in this section, and we discuss the `resample` construct in detail in Sections 3.3 and 6.

We use common syntactic sugar throughout the paper. Most importantly, we use *false* and *true* as aliases for 0 and 1, respectively, and () (unit) as another alias for 0. Furthermore, we often write $g \in \mathbb{G}$ as infix operators. For instance, $1+2$ is a valid term, where $+ \in \mathbb{G}$. Now, let $\mathbb{R}_+$ denote the non-negative reals. We define $f_D : \mathbb{R}^{|D|+1} \to \mathbb{R}_+$ as the function $f_D \in \mathbb{G}$ such that $f_D(c_1,\ldots,c_{|D|},\cdot)$ is the probability *density* (continuous distribution) or *mass* function (discrete distribution) for the probability distribution corresponding to $D \in \mathbb{D}$ and $(c_1,\ldots,c_{|D|})$. For example, $f_{\mathcal{N}}(0,1,x) = \frac{1}{\sqrt{2\pi}} \cdot e^{-\frac{1}{2}\cdot x^2}$ is the standard probability density of the normal distribution with mean 0 and variance 1. Lastly, we will also use `let` bindings, `let rec` bindings, sequencing using `;`, and lists (all of which can be encoded in the calculus). Sequencing is required for the side-effects produced by `weight` (see Definition 5) and `resample` (see Sections 3.3 and 6).

We now consider an example. In Sections 3.2 and 4.3 this example will be further considered to illustrate the semantics, and target measure, respectively. Here, we first give the syntax, and informally visualize the probability distributions (i.e., the target measures, as we will see in Section 4.3) for the example.

Consider first the program in Fig. 2a, directly encoding the *Beta*(2, 2) distribution, illustrated in Fig. 2c. This distribution naturally represents the uncertainty in the bias of a coin—in this case, the coin is most likely unbiased (bias 0.5), and biases closer to 0 and 1 are less likely. In Fig. 2b, we extend Fig. 2a by observing the sequence $[true, false, true]$ when flipping the coin. These observations are encoded using the `weight` construct, which simply accumulates a product (as a side-effect) of all real-valued arguments given to it throughout the execution. First, recall the standard mass function ($\sigma_{f_{Bern}}(p, true) = p$; $\sigma_{f_{Bern}}(p, false) = (1-p)$; $\sigma_{f_{Bern}}(p, x) = 0$ otherwise) for the Bernoulli distribution corresponding to $f_{Bern} \in \mathbb{G}$. The observations $[true, false, true]$ are encoded using the *observe* function, which uses the `weight` construct internally to assign weights to the current value $p$ according to the Bernoulli mass function. As an example, assume we have drawn $p = 0.4$. The weight for this execution is $\sigma_{f_{Bern}}(0.4, true) \cdot \sigma_{f_{Bern}}(0.4, false) \cdot \sigma_{f_{Bern}}(0.4, true) = 0.4^2 \cdot 0.6$. Now consider $p = 0.6$ instead. For this value of $p$ the weight is instead $0.6^2 \cdot 0.4$. This explains the shift in Fig. 2c—a bias closer to 1 is more likely, since we have observed two *true* flips, but only one *false*.

#### 3.2 Semantics

In this section, we define the semantics of our calculus. The definition is split into two parts: a *deterministic semantics* and a *stochastic semantics*. We use evaluation contexts to assist in defining our semantics. The evaluation contexts $\mathbf{E}$ induce a call-by-value semantics, and are defined as follows.

**Definition 2.**

$$\begin{aligned}\mathbf{E} ::= {} & [\cdot] \mid \mathbf{E}\ \mathbf{t} \mid (\lambda x.\mathbf{t})\ \mathbf{E} \mid \mathtt{if}\ \mathbf{E}\ \mathtt{then}\ \mathbf{t}\ \mathtt{else}\ \mathbf{t} \\ & \mid g(c_1, \ldots, c_m, \mathbf{E}, \mathbf{t}_{m+2}, \ldots, \mathbf{t}_{|g|}) \\ & \mid \mathtt{sample}_D(c_1, \ldots, c_m, \mathbf{E}, \mathbf{t}_{m+2}, \ldots, \mathbf{t}_{|D|}) \mid \mathtt{weight}(\mathbf{E})\end{aligned} \tag{2}$$

*We denote the set of all evaluation contexts by* $\mathbb{E}$*.*

With the evaluation contexts in place, we proceed to define the *deterministic semantics* through a small-step relation $\rightarrow_{\text{DET}}$.

**Definition 3.**

$$\frac{}{\mathbf{E}[(\lambda x.\mathbf{t})\ \mathbf{v}] \rightarrow_{\text{DET}} \mathbf{E}[[x \mapsto \mathbf{v}]\mathbf{t}]}(\text{App}) \qquad \frac{c = \sigma_g(c_1, \ldots, c_{|g|})}{\mathbf{E}[g(c_1, \ldots, c_{|g|})] \rightarrow_{\text{DET}} \mathbf{E}[c]}(\text{Prim})$$

$$\frac{}{\mathbf{E}[\mathtt{if}\ true\ \mathtt{then}\ \mathbf{t}_1\ \mathtt{else}\ \mathbf{t}_2] \rightarrow_{\text{DET}} \mathbf{E}[\mathbf{t}_1]}(\text{IfTrue}) \tag{3}$$

$$\frac{}{\mathbf{E}[\mathtt{if}\ false\ \mathtt{then}\ \mathbf{t}_1\ \mathtt{else}\ \mathbf{t}_2] \rightarrow_{\text{DET}} \mathbf{E}[\mathbf{t}_2]}(\text{IfFalse})$$

The rules are straightforward, and will not be discussed in further detail here. We use the standard notation for transitive and reflexive closures (e.g. $\rightarrow^*_{\text{DET}}$), and transitive closures (e.g. $\rightarrow^+_{\text{DET}}$) of relations throughout the paper.

Following the tradition of Kozen [18] and Park et al. [27], sampling in our stochastic semantics works by consuming randomness from a tape of real numbers. We use inverse transform sampling, and therefore the tape consists of numbers from the interval $[0, 1]$. In order to use inverse transform sampling, we require that for each $D \in \mathbb{D}$, there exists a measurable function $F_D^{-1} : \mathbb{R}^{|D|} \times [0, 1] \to \mathbb{R}$, such that $F_D^{-1}(c_1, \ldots, c_{|D|}, \cdot)$ is the *inverse cumulative distribution function* for the probability distribution corresponding to $D$ and $(c_1, \ldots, c_{|D|})$. We call the tape of real numbers a *trace*, and make the following definition.

**Definition 4.** *Let* $\mathbb{N}_0 = \mathbb{N} \cup \{0\}$. *The set of all traces is* $\mathbb{S} = \bigcup_{n \in \mathbb{N}_0} [0, 1]^n$.

We use the notation $(c_1, c_2, \ldots, c_n)_{\mathbb{S}}$ to indicate the trace consisting of the $n$ numbers $c_1, c_2, \ldots, c_n$. Given a trace $s$, we denote by $|s|$ the length of the trace. We also denote the concatenation of two traces $s$ and $s'$ with $s * s'$. Lastly, we let $c :: s$ denote the extension of the trace $s$ with the real number $c$ as head.

With the traces and $F_D^{-1}$ defined, we can proceed to the stochastic[6] semantics $\to$ over $\mathbb{T} \times \mathbb{R}_+ \times \mathbb{S}$.

**Definition 5.**

$$\mathbf{t}_{stop} ::= \mathbf{v} \mid \mathbf{E}[\texttt{sample}_D(c_1, \ldots, c_{|D|})] \mid \mathbf{E}[\texttt{weight}(c)] \mid \mathbf{E}[\texttt{resample}] \tag{4}$$

$$\frac{\mathbf{t} \to_{\text{DET}}^{+} \mathbf{t}_{stop}}{\mathbf{t}, w, s \to \mathbf{t}_{stop}, w, s}(\text{Det}) \quad \frac{c \geq 0}{\mathbf{E}[\texttt{weight}(c)], w, s \to \mathbf{E}[()], w \cdot c, s}(\text{Weight})$$

$$\frac{c = F_D^{-1}(c_1, \ldots, c_{|D|}, p)}{\mathbf{E}[\texttt{sample}_D(c_1, \ldots, c_{|D|})], w, p :: s \to \mathbf{E}[c], w, s}(\text{Sample}) \tag{5}$$

$$\frac{}{\mathbf{E}[\texttt{resample}], w, s \to \mathbf{E}[()], w, s}(\text{Resample})$$

The rule (Det) encapsulates the $\to_{\text{DET}}$ relation, and states that terms can move deterministically only to terms of the form $\mathbf{t}_{stop}$. Note that terms of the form $\mathbf{t}_{stop}$ are found at the left-hand side in the other rules. The (Sample) rule describes how random values are drawn from the inverse cumulative distribution functions and the trace when terms of the form $\texttt{sample}_D(c_1, \ldots, c_{|D|})$ are encountered. Similarly, the Weight rule determines how the weight is updated when $\texttt{weight}(c)$ terms are encountered. Finally, the `resample` construct always evaluates to unit, and is therefore meaningless from the perspective of this semantics. We elaborate on the role of the `resample` construct in Section 3.3.

With the semantics in place, we define two important functions over $\mathbb{S}$ for a given term. In the below definition, assume that a fixed term $\mathbf{t}$ is given.

**Definition 6.**

$$r_{\mathbf{t}}(s) = \begin{cases} \mathbf{v} & \textit{if } \mathbf{t}, 1, s \to^* \mathbf{v}, w, ()_{\mathbb{S}} \\ () & \textit{otherwise} \end{cases} \qquad f_{\mathbf{t}}(s) = \begin{cases} w & \textit{if } \mathbf{t}, 1, s \to^* \mathbf{v}, w, ()_{\mathbb{S}} \\ 0 & \textit{otherwise} \end{cases} \tag{6}$$

[6] Note that the semantics models stochastic behavior, but is itself a deterministic relation.

Intuitively, $r_{\mathbf{t}}$ is the function returning the *result value* after having repeatedly applied $\rightarrow$ on the initial trace $s$. Analogously, $f_{\mathbf{t}}$ gives the *density* or *weight* of a particular $s$. Note that, if $(\mathbf{t}, 1, s)$ gets stuck or diverges, the result value is $()$, and the weight is 0. In other words, we disregard such traces entirely, since we are in practice only interested in probability distributions over values. Furthermore, note that if the final $s \neq ()_{\mathbb{S}}$, the value and weight are again $()$ and 0, respectively. The motivation for this is discussed in Section 4.3.

To illustrate $r_{\mathbf{t}}$, $f_{\mathbf{t}}$, and the `weight` construct, consider the program $\mathbf{t}_{obs}$ in Fig. 2b, and the singleton trace $(0.8)_{\mathbb{S}}$. This program will, in total, evaluate one call to `sample`, and three calls to `weight`. Now, let $h(c) = F^{-1}_{Beta}(2, 2, c)$ and recall the function $\sigma_{f_{Bern}}$ from Section 3.1. Using the notation $\phi(c, x) = \sigma_{f_{Bern}}(h(c), x)$, we have, for some evaluation contexts $\mathbf{E}_1, \mathbf{E}_2, \mathbf{E}_3, \mathbf{E}_4$,

$$
\begin{aligned}
&\mathbf{t}_{obs}, 1, (0.8)_{\mathbb{S}} = \mathbf{E}_1[\mathtt{sample}_{Beta}(2, 2)], 1, (0.8)_{\mathbb{S}} \rightarrow \mathbf{E}_1[h(0.8)], 1, ()_{\mathbb{S}} \\
&\rightarrow \mathbf{E}_2[\mathtt{weight}(\phi(0.8, \mathit{true}))], 1, ()_{\mathbb{S}} \rightarrow \mathbf{E}_2[()], \phi(0.8, \mathit{true}), ()_{\mathbb{S}} \\
&= \mathbf{E}_2[()], h(0.8), ()_{\mathbb{S}} \rightarrow^+ \mathbf{E}_3[()], \phi(0.8, \mathit{false}) \cdot h(0.8), ()_{\mathbb{S}} \\
&\rightarrow^+ \mathbf{E}_4[()], \phi(0.8, \mathit{true}) \cdot (1 - h(0.8)) \cdot h(0.8), ()_{\mathbb{S}} \\
&\rightarrow^+ h(0.8), h(0.8) \cdot (1 - h(0.8)) \cdot h(0.8), ()_{\mathbb{S}}.
\end{aligned}
\tag{7}
$$

That is, $r_{\mathbf{t}_{obs}}((0.8)_{\mathbb{S}}) = h(0.8)$ and $f_{\mathbf{t}_{obs}}((0.8)_{\mathbb{S}}) = h(0.8)^2(1 - h(0.8))$. For arbitrary $c$, we see that $r_{\mathbf{t}_{obs}}((c)_{\mathbb{S}}) = h(c)$ and $f_{\mathbf{t}_{obs}}((c)_{\mathbb{S}}) = h(c)^2(1 - h(c))$. For any other trace $s$ with $|s| \neq 1$, $r_{\mathbf{t}_{obs}}(s) = ()$ and $f_{\mathbf{t}_{obs}}(s) = 0$. We will apply this result when reconsidering this example in Section 4.3.

#### 3.3 Resampling Semantics

In order to connect SMC in PPLs to the classical formalization of SMC presented in Section 5—and thus enabling the theoretical treatments in Sections 6 and 7—we need a relation in which terms "stop" after a certain number $n$ of encountered `resample` terms. In this section, we define such a relation, denoted by $\hookrightarrow$. Its definition is given below.

**Definition 7.**

$$
\frac{\mathbf{t} \neq \mathbf{E}[\mathtt{resample}] \quad \mathbf{t}, w, s \rightarrow \mathbf{t}', w', s'}{\mathbf{t}, w, s, n \hookrightarrow \mathbf{t}', w', s', n} (\textsc{Stoch-Fin})
$$

$$
\frac{n > 0 \quad \mathbf{E}[\mathtt{resample}], w, s \rightarrow \mathbf{E}[()], w, s}{\mathbf{E}[\mathtt{resample}], w, s, n \hookrightarrow \mathbf{E}[()], w, s, n-1} (\textsc{Resample-Fin}) \tag{8}
$$

This relation is $\rightarrow$ extended with a natural number $n$, indicating how many further `resample` terms can be evaluated. We implement this limitation by replacing the rule (Resample) of $\rightarrow$ with (Resample-Fin) of $\hookrightarrow$ above which decrements $n$ each time it is applied, causing terms to get stuck at the $n + 1$th resample encountered.

Now, assume that a fixed term $\mathbf{t}$ is given. We define $r_{\mathbf{t},n}$ and $f_{\mathbf{t},n}$ similar to $r_{\mathbf{t}}$ and $f_{\mathbf{t}}$.

**Definition 8.** $r_{\mathbf{t},n}(s) = \begin{cases} \mathbf{v} & \textit{if } \mathbf{t}, 1, s, n \hookrightarrow^* \mathbf{v}, w, ()_{\mathbb{S}}, n' \\ \mathbf{E}[\texttt{resample}] & \textit{if } \mathbf{t}, 1, s, n \hookrightarrow^* \mathbf{E}[\texttt{resample}], w, ()_{\mathbb{S}}, 0 \\ () & \textit{otherwise} \end{cases}$

**Definition 9.** $f_{\mathbf{t},n}(s) = \begin{cases} w & \textit{if } \mathbf{t}, 1, s, n \hookrightarrow^* \mathbf{v}, w, ()_{\mathbb{S}}, n' \\ w & \textit{if } \mathbf{t}, 1, s, n \hookrightarrow^* \mathbf{E}[\texttt{resample}], w, ()_{\mathbb{S}}, 0 \\ 0 & \textit{otherwise} \end{cases}$

As for $r_{\mathbf{t}}$ and $f_{\mathbf{t}}$, these functions return the result value and weight, respectively, after having repeatedly applied $\hookrightarrow$ on the initial trace $s$. There is one difference compared to $\rightarrow$: besides values, we now also allow stopping with non-zero weight at terms of the form $\mathbf{E}[\texttt{resample}]$.

To illustrate $\hookrightarrow$, $r_{\mathbf{t},n}(s)$, and $f_{\mathbf{t},n}(s)$, consider the term $\mathbf{t}_{seq}$ defined by

$$\begin{array}{l} \texttt{let } \textit{observe } x\ o \texttt{ = weight}(f_{\mathcal{N}}(x,4,o))\texttt{; resample in} \\ \texttt{let } \textit{sim } x_{n-1}\ o_n \texttt{ =} \\ \quad \texttt{let } x_n \texttt{ = sample}_{\mathcal{N}}(x_{n-1}+2,1) \texttt{ in } \textit{observe } x_n\ o_n\texttt{; } x_n \texttt{ in} \\ \texttt{let } x_0 \texttt{ = sample}_{\mathcal{U}}(0,100) \texttt{ in} \\ \texttt{let } f \texttt{ = } \textit{foldl sim} \texttt{ in } f\ x_0\ \texttt{[}c_1, c_2, \ldots, c_{t-1}, c_t\texttt{]}. \end{array} \tag{9}$$

This term encodes a model in which an object moves along a real-valued axis in discrete time steps, but where the actual positions ($x_1$, $x_2$, ...) can only be observed through a noisy sensor ($c_1$, $c_2$, ...). The inference problem consists of finding the probability distribution for the very last position, $x_t$, given all collected observations ($c_1$, $c_2$, ..., $c_t$). Most importantly, note the position of `resample` in (9)—it is evaluated just after evaluating `weight` in every folding step. Because of this, for $n < t$ and all traces $s$ such that $f_{\mathbf{t}_{seq},n}(s) > 0$, we have $r_{\mathbf{t}_{seq},n}(s) = \mathbf{E}^n_{seq}[\texttt{resample; } x_n]$, where $\mathbf{E}^n_{seq} \texttt{ = } f\ [\cdot]\ \texttt{[}c_{n+1}, c_{n+2}, \ldots, c_{t-1}, c_t\texttt{]}$ and where $x_n$ is the value sampled in *sim* at the $n$th folding step. That is, we can now "stop" evaluation at `resample`s. We will revisit this example in Section 6.

### 4 The Target Measure of a Program

In this section, we define the *target measure* induced by any given program in our calculus. We assume basic familiarity with measure theory, Lebesgue integration, and Borel spaces. McDonald and Weiss [23] provide a pedagogical introduction to the subject. In order to define the target measure of a program as a Lebesgue integral (Section 4.3), we require a *measure space* on traces (Section 4.1), and a *measurable space* on terms (Section 4.2). For illustration, we derive the target measure for the example program from Section 3 in Section 4.3. The concepts presented in this section are quite standard, and experienced readers might want to quickly skim it, or even skip it entirely.

#### 4.1 A Measure Space over Traces

We use a standard measure space over traces of samples [22]. First, we define a measurable space over traces. We denote the Borel $\sigma$-algebra on $\mathbb{R}^n$ with $\mathcal{B}^n$, and the Borel $\sigma$-algebra on $[0,1]$ with $\mathcal{B}^n_{[0,1]}$.

**Definition 10.** *The $\sigma$-algebra $\mathcal{S}$ on $\mathbb{S}$ is the $\sigma$-algebra consisting of sets of the form $S = \bigcup_{n\in\mathbb{N}_0} B_n$ with $B_n \in \mathcal{B}^n_{[0,1]}$. Naturally, $[0,1]^0$ is the singleton set containing the empty trace. In other words, $([0,1]^0, \mathcal{B}^0_{[0,1]}) = (\{()_\mathbb{S}\}, \{\{()_\mathbb{S}\}, \emptyset\})$, where $()_\mathbb{S}$ denotes the empty trace.*

**Lemma 1.** $(\mathbb{S}, \mathcal{S})$ *is a measurable space.*$^\dagger$

The most common measure on $\mathcal{B}^n$ is the $n$-dimensional Lebesgue measure, denoted $\lambda_n$. For $n = 0$, we let $\lambda_0 = \delta_{()_\mathbb{S}}$, where $\delta$ denotes the standard Dirac measure. By combining the Lebesgue measures for each $n$, we construct a measure $\mu_\mathbb{S}$ over $(\mathbb{S}, \mathcal{S})$.

**Definition 11.** $\mu_\mathbb{S}(S) = \mu_\mathbb{S}\left(\bigcup_{n\in\mathbb{N}_0} B_n\right) = \sum_{n\in\mathbb{N}_0} \lambda_n(B_n)$

**Lemma 2.** $(\mathbb{S}, \mathcal{S}, \mu_\mathbb{S})$ *is a measure space. Furthermore, $\mu_\mathbb{S}$ is $\sigma$-finite.*$^\dagger$

A comment on notation: we denote universal sets by blackboard bold capital letters (e.g., $\mathbb{S}$), $\sigma$-algebras by calligraphic capital letters (e.g., $\mathcal{S}$), members of $\sigma$-algebras by capital letters (e.g., $S$), and individual elements by lower case letters (e.g., $s$).

#### 4.2 A Measurable Space over Terms

In order to show that $r_\mathbf{t}$ is measurable, we need a measurable space over terms. We let $(\mathbb{T}, \mathcal{T})$ denote the measurable space that we seek to construct, and follow the approach in Staton et al. [35] and Vákár et al. [39]. Because our calculus includes the reals, we would like to at least have $\mathcal{B} \subset \mathcal{T}$. Furthermore, we would also like to extend the Borel measurable sets $\mathcal{B}^n$ to terms with $n$ reals as subterms. For instance, we want sets of the form $\{(\lambda x.\ (\lambda y.\ x + y)\ \ c_2)\ \ c_1 \mid (c_1, c_2) \in B_2\}$ to be measurable, where $B_2 \in \mathcal{B}^2$. This leads us to consider terms in a language in which constants (i.e., reals) are replaced with placeholders $[\cdot]$.

**Definition 12.** *Let $\mathbf{v}_p ::= [\cdot] \mid \lambda x.\mathbf{t}$ replace the values $\mathbf{v}$ from Definition 1. The set of all terms in the resulting new calculus is denoted with $\mathbb{T}_p$.*

Most importantly, it is easy to verify that $\mathbb{T}_p$ is countable. Next, we make the following definitions.

**Definition 13.** *For $n \in \mathbb{N}_0$, we denote by $\mathbb{T}^n_p \subset \mathbb{T}_p$ the set of all terms with exactly $n$ placeholders.*

**Definition 14.** *We let $\mathbf{t}^n_p$ range over the elements of $\mathbb{T}^n_p$. The $\mathbf{t}^n_p$ can be regarded as functions $\mathbf{t}^n_p : \mathbb{R}^n \to \mathbf{t}^n_p(\mathbb{R}_n)$ which replaces the $n$ placeholders with the $n$ reals given as arguments.*

**Definition 15.** $\mathcal{T}_{\mathbf{t}_p^n} = \{\mathbf{t}_p^n(B_n) \mid B_n \in \mathcal{B}^n\}$.

From the above definitions, we construct the required $\sigma$-algebra $\mathcal{T}$.

**Definition 16.** *The $\sigma$-algebra $\mathcal{T}$ on $\mathbb{T}$ is the $\sigma$-algebra consisting of sets of the form $T = \bigcup_{n \in N_0} \bigcup_{\mathbf{t}_p^n \in \mathbb{T}_p^n} \mathbf{t}_p^n(B_n)$.*

**Lemma 3.** *$(\mathbb{T}, \mathcal{T})$ is a measurable space.*$^\dagger$

#### 4.3 The Target Measure

We are now in a position to define the target measure. We will first give the formal definitions, and then illustrate the definitions with an example. The definitions rely on the following result.

**Lemma 4.** *$r_{\mathbf{t}} : (\mathbb{S}, \mathcal{S}) \to (\mathbb{T}, \mathcal{T})$ and $f_{\mathbf{t}} : (\mathbb{S}, \mathcal{S}) \to (\mathbb{R}_+, \mathcal{B}_+)$ are measurable.*$^\dagger$

We can now proceed to define the measure $\langle\!\langle \mathbf{t} \rangle\!\rangle$ over $\mathbb{S}$ induced by a term $\mathbf{t}$ using Lebesgue integration.

**Definition 17.** $\langle\!\langle \mathbf{t} \rangle\!\rangle(S) = \int_S f_{\mathbf{t}}(s)\ d\mu_{\mathbb{S}}(s)$

Using Definition 17 and the measurability of $r_{\mathbf{t}}$, we can also define a corresponding pushforward measure $[\![\mathbf{t}]\!]$ over $\mathbb{T}$.

**Definition 18.** $[\![\mathbf{t}]\!](T) = \langle\!\langle \mathbf{t} \rangle\!\rangle(r_{\mathbf{t}}^{-1}(T)) = \int_{r_{\mathbf{t}}^{-1}(T)} f_{\mathbf{t}}(s)\ d\mu_{\mathbb{S}}(s)$.

The measure $[\![\mathbf{t}]\!]$ is our *target measure*, i.e., the measure encoded by our program that we are interested in.

Let us now consider the target measure for the program given by $\mathbf{t}_{obs}$. It is not too difficult to show that $[\![\mathbf{t}_{obs}]\!](T) = \int_{T \cap \mathbb{R}} c^3(1-c)^2\ d\lambda(c)$. We recognize the integrand as the density for the *Beta*$(4, 3)$ distribution, which, as expected, is exactly the graph shown in Fig. 2c.

We should in some way ensure the target measure is finite (i.e., can be normalized to a probability measure), since we are in the end most often only interested in probability measures. Unfortunately, as observed by Staton [34], there is no known useful syntactic restriction that enforces finite measures in PPLs while still admitting weights $> 1$. We will discuss this further in Section 6.2 in relation to SMC in our calculus.

Lastly, from Section 3.2, recall that we disallow non-empty final traces in $f_{\mathbf{t}}$ and $r_{\mathbf{t}}$. We see here why this is needed: if allowed, for every trace $s$ with $f_{\mathbf{t}}(s) > 0$, *all* extensions $s * s'$ have the same density $f_{\mathbf{t}}(s * s') = f_{\mathbf{t}}(s) > 0$. From this, it is easy to check that if $[\![\mathbf{t}]\!] \neq 0$ (the zero measure), then $[\![\mathbf{t}]\!](\mathbb{T}) = \infty$ (i.e., the measure is not finite). In fact, for any $T \in \mathcal{T}$, $[\![\mathbf{t}]\!](T) > 0 \implies [\![\mathbf{t}]\!](T) = \infty$. Clearly, this is not a useful target measure.

## 5 Formal SMC

In this section, we give a generic formalization of SMC based on Chopin [6]. We assume a basic understanding of SMC. For a complete introduction to SMC, we recommend Naesseth et al. [26] and Doucet and Johansen [10].

First, in Section 5.1, we introduce transition kernels, which is a fundamental concept used in the remaining sections of the paper. Second, in Section 5.2, we describe Chopin's generic formalization of SMC as an algorithm for approximating a sequence of distributions based on a sequence of approximating transition kernels. Lastly, in Section 5.3, we give standard correctness results for the algorithm.

### 5.1 Preliminaries: Transition Kernels

Intuitively, transition kernels describe how elements move between measurable spaces. For a more comprehensive introduction, see Vákár and Ong [40].

**Definition 19.** *Let* $(\mathbb{A}, \mathcal{A})$ *and* $(\mathbb{A}', \mathcal{A}')$ *be measurable spaces, and let* $\mathcal{B}_+^* = \{B \mid B \setminus \{\infty\} \in \mathcal{B}_+\}$. *A function* $k : \mathbb{A} \times \mathcal{A}' \to \mathbb{R}_+^*$ *is a* (transition) kernel *if (1) for all* $a \in \mathbb{A}$, $k(a, \cdot) : \mathcal{A}' \to \mathbb{R}_+^*$ *is a measure on* $\mathcal{A}'$, *and (2) for all* $A' \in \mathcal{A}'$, $k(\cdot, A') : (\mathbb{A}, \mathcal{A}) \to (\mathbb{R}_+^*, \mathcal{B}_+^*)$ *is measurable.*

Additionally, we can classify transition kernels according to the below definition.

**Definition 20.** *Let* $(\mathbb{A}, \mathcal{A})$ *and* $(\mathbb{A}', \mathcal{A}')$ *be measurable spaces. A kernel* $k : \mathbb{A} \times \mathcal{A}' \to \mathbb{R}_+^*$ *is a* sub-probability kernel *if* $k(a, \cdot)$ *is a sub-probability measure for all* $a \in \mathbb{A}$*; a* probability kernel *if* $k(a, \cdot)$ *is a probability measure for all* $a \in \mathbb{A}$*; and a* finite kernel *if* $\sup_{a \in \mathbb{A}} k(a, \mathbb{A}') < \infty$.

### 5.2 Algorithm

The starting point in Chopin's formulation of SMC is a sequence of probability measures $\pi_n$ (over respective measurable spaces $(\mathbb{A}_n, \mathcal{A}_n)$, with $n \in \mathbb{N}_0$) that are difficult or impossible to directly draw samples from.

The SMC approach is to generate samples from the $\pi_n$ by first sampling from a sequence of *proposal measures* $q_n$, and then correcting for the discrepancy between these measures by weighting the proposal samples. The proposal distributions are generated from an initial measure $q_0$ and a sequence of transition kernels $k_n : \mathbb{A}_{n-1} \times \mathcal{A}_n \to [0, 1], n \in \mathbb{N}$ as

$$q_n(A_n) = \int_{\mathbb{A}_{n-1}} k_n(a_{n-1}, A_n) \, d\pi_{n-1}(a_{n-1}). \tag{10}$$

In order to approximate $\pi_n$ by weighting samples from $q_n$, we need some way of obtaining the appropriate weights. Hence, we require each measurable space $(\mathbb{A}_n, \mathcal{A}_n)$ to have a default $\sigma$-finite measure $\mu_{\mathbb{A}_n}$, and the measures $\pi_n$ and $q_n$ to

**Algorithm 1** A generic formulation of sequential Monte Carlo inference based on Chopin [6]. In each step, we let $1 \leq j \leq J$, where $J$ is the number of samples.

1. **Initialization:** Set $n = 0$. Draw $a_0^j \sim q_0$ for $1 \leq j \leq J$.
   The empirical distribution given by $\{a_0^j\}_{j=1}^J$ approximates $q_0$.
2. **Correction:** Calculate $w_n^j = \frac{f_{\widetilde{\pi}_n}(a_n^j)}{f_{\widetilde{q}_n}(a_n^j)}$.
   The empirical distribution given by $\{(a_n^j, w_n^j)\}_{j=1}^J$ approximates $\pi_n$.
3. **Selection:** Resample the empirical distribution $\{(a_n^j, w_n^j)\}_{j=1}^J$.
   The new empirical distribution is unweighted and is given by $\{\hat{a}_n^j\}_{j=1}^J$. This distribution also approximates $\pi_n$.
4. **Mutation:** Increment $n$.
   Draw $a_n^j \sim k_n(\hat{a}_{n-1}^j, \cdot)$ for $1 \leq j \leq J$. The empirical distribution given by $\{a_n^j\}_{j=1}^J$ approximates $q_n$. Go to (2).

have densities $f_{\pi_n}$ and $f_{q_n}$ with respect to this default measure. Furthermore, we require that the functions $f_{\pi_n}$ and $f_{q_n}$ can be efficiently computed pointwise, up to an unknown constant factor per function and value of $n$. More precisely, we can efficiently compute the densities $f_{\widetilde{\pi}_n} = Z_{\widetilde{\pi}_n} \cdot f_{\pi_n}$ and $f_{\widetilde{q}_n} = Z_{\widetilde{q}_n} \cdot f_{q_n}$, corresponding to the unnormalized measures $\widetilde{\pi}_n = Z_{\widetilde{\pi}_n} \cdot \pi_n$ and $\widetilde{q}_n = Z_{\widetilde{q}_n} \cdot q_n$. Here, $Z_{\widetilde{\pi}_n} = \widetilde{\pi}_n(\mathbb{A}_n) \in \mathbb{R}_+$ and $Z_{\widetilde{q}_n} = \widetilde{q}_n(\mathbb{A}_n) \in \mathbb{R}_+$ denote the unknown *normalizing constants* for the distributions $\widetilde{\pi}_n$ and $\widetilde{q}_n$.

Algorithm 1 presents a generic version of SMC [6] for approximating $\pi_n$. We make the notion of approximation used in the algorithm precise in Section 5.3. Note that in the correction step, the unnormalized pointwise evaluation of $f_{\pi_n}$ and $f_{q_n}$ is used to calculate the weights. In the algorithm description, we also use some new terminology. First, an *empirical distribution* is the discrete probability measure formed by a finite set of possibly weighted samples $\{(a_n^j, w_n^j)\}_{j=1}^J$, where $a_n^j \in \mathbb{A}_n$ and $w_n^j \in \mathbb{R}_+$. Second, when *resampling* an empirical distribution, we sample $J$ times from it (with replacement), with each sample having its normalized weight as probability of being sampled. More specifically, this is known as *multinomial resampling*. Other resampling schemes also exist [8], and are often used in practice to reduce variance. After resampling, the set of samples forms a new empirical distribution with $J$ unweighted (all $w_n^j = 1$) samples.

An important feature of SMC compared to other inference algorithms is that SMC produces, as a by-product of inference, unbiased estimates $\hat{Z}_{\widetilde{\pi}_n}$ of the normalizing constants $Z_{\widetilde{\pi}_n}$. Stated differently, this means that Algorithm 1 not only approximates the $\pi_n$, but also the unnormalized versions $\widetilde{\pi}_n$. From the weights $w_n^j$ in Algorithm 1, the estimates are given by

$$\hat{Z}_{\widetilde{\pi}_n} = \prod_{i=0}^{n} \frac{1}{J} \sum_{j=1}^{J} w_i^j \approx Z_{\widetilde{\pi}_n} \tag{11}$$

for each $\widetilde{\pi}_n$. We give the unbiasedness result of $\hat{Z}_{\widetilde{\pi}_n}$ in Lemma 5 (item 2) below. The normalizing constant is often used to compare the accuracy of different probabilistic models, and as such, it is also known as the *marginal likelihood*, or *model evidence*. For an example application, see Ronquist et al. [30].

To conclude this section, note that many sequences of probability kernels $k_n$ can be used to approximate the same sequence of measures $\pi_n$. The only requirement on the $k_n$ is that $f_{\pi_n}(a_n) > 0 \implies f_{q_n}(a_n) > 0$ must hold for all $n \in \mathbb{N}_0$ and $a_n \in \mathbb{A}_n$ (i.e., the proposals must "cover" the $\pi_n$) [9]. We call such a sequence of kernels $k_n$ *valid*. Different choices of $k_n$ induce different proposals $q_n$, and hence capture different SMC algorithms. The most common example is the BPF, which directly uses the kernels from the model as the sequence of kernels in the SMC algorithm (hence the "bootstrap"). In Section 7.1, we formalize the bootstrap kernels in the context of our calculus. However, we may want to choose other probability kernels that satisfy the covering condition, since the choice of kernels can have major implications for the rate of convergence [28].

#### 5.3 Correctness

We begin by defining the notion of approximation used in Algorithm 1.

**Definition 21 (Based on Chopin [6, p. 2387]).** *Let* $(\mathbb{A}, \mathcal{A})$ *denote a measurable space,* $\{\{(a^{j,J}, w^{j,J})\}_{j=1}^{J}\}_{J\in\mathbb{N}}$ *a triangular array of random variables in* $\mathbb{A} \times \mathbb{R}$*, and* $\pi : \mathcal{A} \to \mathbb{R}_+^*$ *a probability measure. We say that* $\{\{(a^{j,J}, w^{j,J})\}_{j=1}^{J}\}_{J\in\mathbb{N}}$ approximates $\pi$ *if the equality* $\lim_{J\to\infty} \frac{\sum_{j=1}^{J} w^{j,J}\varphi(a^{j,J})}{\sum_{j=1}^{J} w^{j,J}} = \mathbb{E}_\pi(\varphi)$ *holds almost surely for all measurable functions* $\varphi : (\mathbb{A}, \mathcal{A}) \to (\mathbb{R}, \mathcal{B})$ *such that* $\mathbb{E}_\pi(\varphi)$*—the expected value of the function* $\varphi$ *over the distribution* $\pi$*—exists.*

First, note that the triangular array can also be viewed as a sequence of random empirical distributions (indexed by $J$). Precisely such sequences are formed by the random empirical distributions in Algorithm 1 when indexed by the increasing number of samples $J$. For simplicity, we often let context determine the sequence, and directly state that a random empirical distribution approximates some distribution (as in Algorithm 1).

Two classical results in SMC literature are given in the following lemma: a law of large numbers and the unbiasedness of the normalizing constant estimate. We take these results as the definition of SMC correctness used in this paper.

**Lemma 5.** *Let* $\pi_n$*,* $n \in \mathbb{N}_0$*, be a sequence of probability measures over measurable spaces* $(\mathbb{A}_n, \mathcal{A}_n)$ *with default σ-finite measures* $\mu_{\mathbb{A}_n}$*, such that the* $\pi_n$ *have densities* $f_{\pi_n}$ *with respect to these default measures. Furthermore, let* $q_0$ *be a probability measure with density* $f_{q_0}$ *with respect to* $\mu_{\mathbb{A}_0}$*, and* $k_n$ *a sequence of probability kernels inducing a sequence of proposal probability measures* $q_n$*, given by* (10)*, over* $(\mathbb{A}_n, \mathcal{A}_n)$ *with densities* $f_{q_n}$ *with respect to* $\mu_{\mathbb{A}_n}$*. Also, assume the* $k_n$ *are valid, i.e., that that* $f_{\pi_n}(a_n) > 0 \implies f_{q_n}(a_n) > 0$ *holds for all* $n \in \mathbb{N}_0$ *and* $a_n \in \mathbb{A}_n$*. Then*

1. *the empirical distributions* $\{(a_n^j, w_n^j)\}_{j=1}^{J}$ *and* $\{\hat{a}_n^j\}_{j=1}^{J}$ *produced by Algorithm 1 approximate* $\pi_n$ *for each* $n \in \mathbb{N}_0$*; and*

2. $\mathbb{E}(\hat{Z}_{\widetilde{\pi}_n}) = Z_{\widetilde{\pi}_n}$ *for each* $n \in \mathbb{N}_0$*, where the expectation is taken with respect to the weights produced when running Algorithm 1, and* $\hat{Z}_{\widetilde{\pi}_n}$ *is given by* (11)*.*

*Proof.* As referenced in Naesseth et al. [26], see Del Moral [7][Theorem 7.4.3] for 1. For 2, see Naesseth et al. [26][Appendix 4.A].

Chopin [6][Theorem 1] gives another SMC convergence result in the form of a central limit. This result, however, requires further restrictions on the weights $w_n^j$ in Algorithm 1. It is not clear when these restrictions are fulfilled when applying SMC on a program in our calculus. This is an interesting topic for future work.

### 6 Formal SMC for Probabilistic Programming Languages

This section contains our main contribution: how to interpret the operational semantics of our calculus as the unnormalized sequence of measures $\widetilde{\pi}_n$ in Chopin's formalization (Section 6.1), as well as sufficient conditions for this sequence of approximating measures to converge to $\langle\!\langle \mathbf{t} \rangle\!\rangle$ and for the normalizing constant estimate to be correct (Section 6.2).

An important insight during this work was that it is more convenient to find an approximating sequence of measures $\langle\!\langle \mathbf{t} \rangle\!\rangle_n$ to the trace measure $\langle\!\langle \mathbf{t} \rangle\!\rangle$, compared to finding a sequence of measures $[\![\mathbf{t}]\!]_n$ directly approximating the target measure $[\![\mathbf{t}]\!]$. In Section 6.1, we define $\langle\!\langle \mathbf{t} \rangle\!\rangle_n$ similarly to $\langle\!\langle \mathbf{t} \rangle\!\rangle$, except that at most $n$ evaluations of `resample` are allowed. This upper bound on the number of `resample`s is formalized through the relation $\hookrightarrow$ from Section 3.3.

In Section 6.2, we obtain two different conditions for the convergence of the sequence $\langle\!\langle \mathbf{t} \rangle\!\rangle_n$ to $\langle\!\langle \mathbf{t} \rangle\!\rangle$: Theorem 1 states that for programs with an upper bound $N$ on the number of `resample`s they evaluate, $\langle\!\langle \mathbf{t} \rangle\!\rangle_N = \langle\!\langle \mathbf{t} \rangle\!\rangle$. This precondition holds in many practical settings, for instance where each resampling is connected to a datum collected before inference starts. Theorem 2 states another convergence result for programs without such an upper bound but with dominated weights. Because of these convergence results, we can often approximate $\langle\!\langle \mathbf{t} \rangle\!\rangle$ by approximating $\langle\!\langle \mathbf{t} \rangle\!\rangle_n$ with Algorithm 1. When this is the case, Lemma 5 implies that Algorithm 1, either after a sufficient number of time steps or asymptotically, correctly approximates $\langle\!\langle \mathbf{t} \rangle\!\rangle$ and the normalizing constant $Z_{\langle\!\langle \mathbf{t} \rangle\!\rangle}$. This is the content of Theorem 3. We conclude Section 6.2 by discussing `resample` placements and their relation to Theorem 3, as well as practical implications of Theorem 3.

#### 6.1 The Sequence of Measures Generated by a Program

We now apply the formalization from Section 4.3 again, but with $f_{\mathbf{t},n}$ and $r_{\mathbf{t},n}$ (from Section 3.3) replacing $f_{\mathbf{t}}$ and $r_{\mathbf{t}}$. Intuitively, this yields a sequence of measures $[\![\mathbf{t}]\!]_n$ indexed by $n$, which are similar to $[\![\mathbf{t}]\!]$, but only allow for evaluating at most $n$ resamples. To illustrate this idea, consider again the program $\mathbf{t}_{seq}$ in (9). Here, $[\![\mathbf{t}_{seq}]\!]_0$ is a distribution over terms of the form $\mathbf{E}^1_{seq}[$`resample;` $x_1]$, $[\![\mathbf{t}_{seq}]\!]_1$ a distribution over terms of the form $\mathbf{E}^2_{seq}[$`resample;` $x_2]$, and so forth.

For $n \geq t$, $[\![\mathbf{t}_{seq}]\!]_n = [\![\mathbf{t}_{seq}]\!]$, because it is clear that $t$ is an upper bound on the number of resamples evaluated in $\mathbf{t}_{seq}$.

While the measures $[\![\mathbf{t}]\!]_n$ are useful for giving intuition, it is easier from a technical perspective to define and work with $\langle\!\langle \mathbf{t} \rangle\!\rangle_n$, the sequence of measures over *traces* where at most $n$ `resample`s are allowed. First, we need the following result, analogous to Lemma 4.

**Lemma 6.** *$r_{\mathbf{t},n} : (\mathbb{S}, \mathcal{S}) \to (\mathbb{T}, \mathcal{T})$ and $f_{\mathbf{t},n} : (\mathbb{S}, \mathcal{S}) \to (\mathbb{R}_+, \mathcal{B}_+)$ are measurable.*†

This allows us to define $\langle\!\langle \mathbf{t} \rangle\!\rangle_n$ (cf. Definition 17).

**Definition 22.** $\langle\!\langle \mathbf{t} \rangle\!\rangle_n(S) = \int_S f_{\mathbf{t},n}(s)\ d\mu_{\mathbb{S}}(s)$

### 6.2 Correctness

We begin with a convergence result for when the number of calls to `resample` in a program is upper bounded.

**Theorem 1.** *If there is $N \in \mathbb{N}$ such that $f_{\mathbf{t},n} = f_{\mathbf{t}}$ whenever $n > N$, then $\langle\!\langle \mathbf{t} \rangle\!\rangle_n = \langle\!\langle \mathbf{t} \rangle\!\rangle$ for all $n > N$.*

This follows directly since $f_{\mathbf{t},n}$ not only converges to $f_{\mathbf{t}}$, but is also equal to $f_{\mathbf{t}}$ for all $n > N$. However, even if the number of calls to `resample` in $\mathbf{t}$ is upper bounded, there is still one concern with using $\langle\!\langle \mathbf{t} \rangle\!\rangle_n$ as $\widetilde{\pi}_n$ in Algorithm 1: there is no guarantee that the measures $\langle\!\langle \mathbf{t} \rangle\!\rangle_n$ can be normalized to probability measures and have unique densities (i.e., that they are finite). This is a requirement for the correctness results in Lemma 5. Unfortunately, recall from Section 4.3 that there is no known useful syntactic restriction that enforces finiteness of the target measure. This is clearly true for the measures $\langle\!\langle \mathbf{t} \rangle\!\rangle_n$ as well, and as such, we need to *make the assumption* that the $\langle\!\langle \mathbf{t} \rangle\!\rangle_n$ are finite—otherwise, it is not clear that Algorithm 1 produces the correct result, since the conditions in Lemma 5 are not fulfilled. Fortunately, this assumption is valid for most, if not all, models of practical interest. Nevertheless, investigating whether or not the restriction to probability measures in Lemma 5 can be lifted to some extent is an interesting topic for future work.

Although of limited practical interest, programs with an unbounded number of calls to `resample` are of interest from a semantic perspective. If we have $\lim_{n\to\infty} \langle\!\langle \mathbf{t} \rangle\!\rangle_n = \langle\!\langle \mathbf{t} \rangle\!\rangle$ pointwise, then any SMC algorithm approximating the sequence $\langle\!\langle \mathbf{t} \rangle\!\rangle_n$ also approximates $\langle\!\langle \mathbf{t} \rangle\!\rangle$, at least asymptotically in the number of steps $n$. First, consider the program $\mathbf{t}_{geo\text{-}res}$ given by

```
let rec geometric _ =
  resample; if sample_bern(0.6) then 1 + geometric () else 1        (12)
in geometric ().
```

Note that $\mathbf{t}_{geo\text{-}res}$ has no upper bound on the number of calls to `resample`, and therefore Theorem 1 is not applicable. It is easy, however, to check that $\lim_{n\to\infty} \langle\!\langle \mathbf{t}_{geo\text{-}res} \rangle\!\rangle_n = \langle\!\langle \mathbf{t}_{geo\text{-}res} \rangle\!\rangle$ pointwise. So does $\lim_{n\to\infty} \langle\!\langle \mathbf{t} \rangle\!\rangle_n = \langle\!\langle \mathbf{t} \rangle\!\rangle$ pointwise hold in general? The answer is no, as we demonstrate next.

For $\lim_{n\to\infty}\langle\!\langle \mathbf{t} \rangle\!\rangle_n = \langle\!\langle \mathbf{t} \rangle\!\rangle$ to hold pointwise, it must hold that $\lim_{n\to\infty} f_{\mathbf{t},n} = f_{\mathbf{t}}$ pointwise $\mu_{\mathbb{S}}$-ae. Unfortunately, this does not hold for all programs. Consider the program $\mathbf{t}_{loop}$ defined by `let rec loop _ = resample; loop () in loop ()`. Here, $f_{\mathbf{t}_{loop}} = 0$ since the program diverges deterministically, but $f_{\mathbf{t}_{loop},n}(()_{\mathbb{S}}) = 1$ for all $n$. Because $\mu_{\mathbb{S}}(\{()_{\mathbb{S}}\}) \neq 0$, we do not have $\lim_{n\to\infty} f_{\mathbf{t}_{loop},n} = f_{\mathbf{t}_{loop}}$ pointwise $\mu_{\mathbb{S}}$-ae.

Even if we have $\lim_{n\to\infty} f_{\mathbf{t},n} = f_{\mathbf{t}}$ pointwise $\mu_{\mathbb{S}}$-ae, we might not have $\lim_{n\to\infty}\langle\!\langle \mathbf{t} \rangle\!\rangle_n = \langle\!\langle \mathbf{t} \rangle\!\rangle$ pointwise. Consider, for instance, the program $\mathbf{t}_{unit}$ given by

```
let s = sample_U(0,1) in
let rec foo n =
  if s ≤ 1/n then resample; weight 2; foo (2·n) else weight 0 in
foo 1
```
(13)

We have $f_{\mathbf{t}_{unit}} = 0$ and $f_{\mathbf{t}_{unit},n} = 2^n \cdot \mathbf{1}_{[0,1/2^n]}$ for $n > 0$. Also, $\lim_{n\to\infty} f_{\mathbf{t}_{unit},n} = f_{\mathbf{t}_{unit}}$ pointwise. However, $\lim_{n\to\infty}\langle\!\langle \mathbf{t}_{unit} \rangle\!\rangle_n(\mathbb{S}) = 1 \neq 0 = \langle\!\langle \mathbf{t}_{unit} \rangle\!\rangle(\mathbb{S})$. This shows that the limit may fail to hold, even for programs that terminate almost surely, as is the case for the program $\mathbf{t}_{unit}$ in (13). In fact, this program is positively almost surely terminating [4] since the expected number of recursive calls to `foo` is 1.

Guided by the previous example, we now state the dominated convergence theorem—a fundamental result in measure theory—in the context of SMC inference in our calculus.

**Theorem 2.** *Assume that* $\lim_{n\to\infty} f_{\mathbf{t},n} = f_{\mathbf{t}}$ *holds pointwise* $\mu_{\mathbb{S}}$*-ae. Furthermore, assume that there exists a measurable function* $g : (\mathbb{S}, \mathcal{S}) \to (\mathbb{R}_+, \mathcal{B}_+)$ *such that* $f_{\mathbf{t},n} \leq g$ $\mu_{\mathbb{S}}$*-ae for all* $n$*, and* $\int_{\mathbb{S}} g(s) d\mu_{\mathbb{S}}(s) < \infty$*. Then* $\lim_{n\to\infty}\langle\!\langle \mathbf{t} \rangle\!\rangle_n = \langle\!\langle \mathbf{t} \rangle\!\rangle$ *pointwise.*

For a proof, see McDonald and Weiss [23, Theorem 4.9]. It is easy to check that for our example in (13), there is no dominating and integrable $g$ as is required in Theorem 2. We have already seen that the conclusion of the theorem fails to hold here. As a corollary, if there exists a dominating and integrable $g$, the measures $\langle\!\langle \mathbf{t} \rangle\!\rangle_n$ are always finite.

**Corollary 1.** *If there exists a measurable function* $g : (\mathbb{S}, \mathcal{S}) \to (\mathbb{R}_+, \mathcal{B}_+)$ *such that* $f_{\mathbf{t},n} \leq g$ $\mu_{\mathbb{S}}$*-ae for all* $n$*, and* $\int_{\mathbb{S}} g(s) d\mu_{\mathbb{S}}(s) < \infty$*, then* $\langle\!\langle \mathbf{t} \rangle\!\rangle_n$ *is finite for each* $n \in \mathbb{N}_0$*.*

This holds because $\langle\!\langle \mathbf{t} \rangle\!\rangle_n(\mathbb{S}) = \int_{\mathbb{S}} f_{\mathbf{t},n}(s) d\mu_{\mathbb{S}}(s) \leq \int_{\mathbb{S}} g(s) d\mu_{\mathbb{S}}(s) < \infty$. Hence, we do not need to assume the finiteness of $\langle\!\langle \mathbf{t} \rangle\!\rangle_n$ in order for Algorithm 1 to be applicable, as was the case for the setting of Theorem 1.

In Theorem 3, we summarize and combine the above results with Lemma 5.

**Theorem 3.** *Let* $\mathbf{t}$ *be a term, and apply Algorithm 1 with* $\langle\!\langle \mathbf{t} \rangle\!\rangle_n$ *as* $\widetilde{\pi}_n$*, and with arbitrary valid kernels* $k_n$*. If the condition of Theorem 1 holds and* $\langle\!\langle \mathbf{t} \rangle\!\rangle_n$ *is finite for each* $n \in \mathbb{N}_0$*, then Algorithm 1 approximates* $\langle\!\langle \mathbf{t} \rangle\!\rangle$ *and its normalizing constant after a finite number of steps. Alternatively, if the condition of Theorem 2*

*holds, then Algorithm 1 approximates* $\langle\!\langle \mathbf{t} \rangle\!\rangle$ *and its normalizing constant in the limit* $n \to \infty$.

This follows directly from Theorem 1, Theorem 2, and Lemma 5.

We conclude this section by discussing `resample` placements, and the practical implications of Theorem 3. First, we define a `resample` *placement* for a term $\mathbf{t}$ as the term resulting from replacing arbitrary subterms $\mathbf{t}'$ of $\mathbf{t}$ with `resample;` $\mathbf{t}'$. Note that such a placement directly corresponds to constructing the sequence $\langle\!\langle \mathbf{t} \rangle\!\rangle_n$. Second, note that the measure $\langle\!\langle \mathbf{t} \rangle\!\rangle$ and the target measure $[\![\mathbf{t}]\!]$ are clearly *unaffected* by such a placement—indeed, `resample` simply evaluates to (), and for $\langle\!\langle \mathbf{t} \rangle\!\rangle$ and $[\![\mathbf{t}]\!]$, there is no bound on how many `resample`s we can evaluate. As such, we conclude that *all* resample placements in $\mathbf{t}$ fulfilling one of the two conditions in Theorem 3 leads to a correct approximation of $\langle\!\langle \mathbf{t} \rangle\!\rangle$ when applying Algorithm 1. Furthermore, there is always, in practice, an upper bound on the number of calls to `resample`, since any concrete run of SMC has an (explicit or implicit) upper bound on its runtime. This is a powerful result, since it implies that when implementing SMC for PPLs, any method for selecting resampling locations in a program is correct under mild conditions (Theorem 1 or Theorem 2) that are most often, if not always, fulfilled in practice. Most importantly, this justifies the basic approach for placing `resample`s found in WebPPL, Anglican, and Birch, in which every call to `weight` is directly followed (implicitly) by a call to `resample`. It also justifies the approach to placing `resample`s described in Lundén et al. [21]. This latter approach is essential in, e.g., Ronquist et al. [30], in order to increase inference efficiency.

Our results also show that the restriction in Anglican requiring all executions to encounter the same number of `resample`s, is too conservative. Clearly, this is not a requirement in either Theorem 1 or Theorem 2. For instance, the number of calls to `resample` varies significantly in (12).

### 7 SMC Algorithms

In this section, we take a look at how the kernels $k_n$ in Algorithm 1 can be instantiated to yield the concrete SMC algorithm known as the bootstrap particle filter (Section 7.1), and also discuss other SMC algorithms and how they relate to Algorithm 1 (Section 7.2).

#### 7.1 The Bootstrap Particle Filter

We define for each term $\mathbf{t}$ a particular sequence of kernels $k_{\mathbf{t},n}$, that gives rise to the SMC algorithm known as the bootstrap particle filter (BPF). Informally, these kernels correspond to simply continuing to evaluate the program until either arriving at a value $\mathbf{v}$ or a term of the form $\mathbf{E}[$`resample`$]$. For the bootstrap kernel, calculating the weights $w_n^j$ from Algorithm 1 is particularly simple.

Similarly to $\langle\!\langle \mathbf{t} \rangle\!\rangle_n$, it is more convenient to define and work with sequences of kernels over traces, rather than terms. We will define $k_{\mathbf{t},n}(s, \cdot)$ to be the sub-probability measure over extended traces $s * s'$ resulting from evaluating the term $r_{\mathbf{t},n-1}(s)$ until the next `resample` or value $\mathbf{v}$, ignoring any call to `weight`. First, we immediately have that the set of all traces that do not have $s$ as prefix must have measure zero. To make this formal, we will use the inverse images of the functions $prepend_s(s') = s * s'$, $s \in \mathbb{S}$ in the definition of the kernel.

**Lemma 7.** *The functions* $prepend_s : (\mathbb{S}, \mathcal{S}) \to (\mathbb{S}, \mathcal{S})$ *are measurable.*†

The next ingredient for defining the kernels $k_{\mathbf{t},n}$ is a function $p_{\mathbf{t},n}$ that indicates what traces are possible when executing $\mathbf{t}$ until the $n+1$th `resample` or value.

**Definition 23.** $p_{\mathbf{t},n}(s) = \begin{cases} 1 & \textit{if } \mathbf{t}, \cdot, s, n \hookrightarrow^* \mathbf{v}, \cdot, ()_{\mathbb{S}}, \cdot \\ 1 & \textit{if } \mathbf{t}, \cdot, s, n \hookrightarrow^* \mathbf{E}[\texttt{resample}], \cdot, ()_{\mathbb{S}}, 0 \\ 0 & \textit{otherwise} \end{cases}$

Note the similarities to Definition 9. In particular, $f_{\mathbf{t},n}(s) > 0$ implies $p_{\mathbf{t},n}(s) = 1$. However, note that $f_{\mathbf{t},n}(s) = 0$ does not imply $p_{\mathbf{t},n}(s) = 0$, since $p_{\mathbf{t},n}$ ignores weights. As an example, $f_{(\texttt{weight}\ 0),n}(()_{\mathbb{S}}) = 0$, while $p_{(\texttt{weight}\ 0),n}(()_{\mathbb{S}}) = 1$.

**Lemma 8.** $p_{\mathbf{t},n} : (\mathbb{S}, \mathcal{S}) \to (\mathbb{R}_+, \mathcal{B}_+)$ *is measurable.*

The proof is analogous to that of Lemma 6. We can now formally define the kernels $k_{\mathbf{t},n}$.

**Definition 24.** $k_{\mathbf{t},n}(s, S) = \int_{prepend_s^{-1}(S)} p_{r_{\mathbf{t},n-1}(s),1}(s')\ d\mu_{\mathbb{S}}(s')$

By the definition of $p_{\mathbf{t},n}$, the $k_{\mathbf{t},n}$ are *sub-probability* kernels rather than probability kernels. Intuitively, the reason for this is that during evaluation, terms can get stuck, deterministically diverge, or even stochastically diverge. Such traces are assigned 0 weight by $p_{\mathbf{t},n}$.

**Lemma 9.** *The functions* $k_{\mathbf{t},n} : \mathbb{S} \times \mathcal{S} \to \mathbb{R}_+$ *are sub-probability kernels.*†[7]

We get a natural starting measure $q_0$ from the sub-probability distribution resulting from running the initial program $\mathbf{t}$ until reaching a value or a call to `resample`, ignoring weights.

**Definition 25.** $\langle \mathbf{t} \rangle_0(S) = \int_S p_{\mathbf{t},0}(s) d\mu_{\mathbb{S}}(s)$.

Now we have all the ingredients for the general SMC algorithm described in Section 5.2: a sequence of target measures $\langle\!\langle \mathbf{t} \rangle\!\rangle_n = \widetilde{\pi}_n$ (Definition 22), a starting measure $\langle \mathbf{t} \rangle_0 \propto q_0$ (Definition 25), and a sequence of kernels $k_{\mathbf{t},n} \propto k_n$ (Definition 24). These then induce a sequence of proposal measures $\langle \mathbf{t} \rangle_n = \widetilde{q}_n$ as in Equation (10), which we instantiate in the following definition.

**Definition 26.** $\langle \mathbf{t} \rangle_n(S) = \int_{\mathbb{S}} k_{\mathbf{t},n}(s, S) f_{\mathbf{t},n-1}(s) d\mu_{\mathbb{S}}(s), \quad n > 0$

Intuitively, the measures $\langle \mathbf{t} \rangle_n$ are obtained by evaluating the terms in the support of the measure $\langle\!\langle \mathbf{t} \rangle\!\rangle_{n-1}$ until reaching the next `resample` or value. For an efficient implementation, we need to factorize this definition into the history and the current step, which amounts to splitting the traces. Each feasible trace can be split in such a way.

[7] We only give a partial proof of this lemma.

**Algorithm 2** A concrete instantiation of Algorithm 1 with $\widetilde{\pi}_n = \langle\!\langle \mathbf{t} \rangle\!\rangle_n$, $k_n \propto k_{\mathbf{t},n}$, $q_0 \propto \langle \mathbf{t} \rangle_0$, and as a consequence $\widetilde{q}_n = \langle \mathbf{t} \rangle_n$ (for $n > 0$). In each step, we let $1 \leq j \leq J$, where $J$ is the number of samples.

1. **Initialization:** Set $n = 0$. Draw $s_0^j \sim \langle \mathbf{t} \rangle_0$ for $1 \leq j \leq J$.
   That is, run the program $\mathbf{t}$, and draw from $\mathcal{U}(0,1)$ whenever required by a $\texttt{sample}_D$. Record these draws as the trace $s_0^j$. Stop when reaching a term of the form $\mathbf{E}[\texttt{resample}]$ or a value $\mathbf{v}$. The empirical distribution $\{s_0^j\}_{j=1}^J$ approximates $\langle \mathbf{t} \rangle_0$.
2. **Correction:** Calculate $w_n^j = \frac{f_{\langle\!\langle \mathbf{t} \rangle\!\rangle_n}(s_n^j)}{f_{\langle \mathbf{t} \rangle_n}(s_n^j)}$ for $1 \leq j \leq J$.
   As a consequence of Lemma 13, this is trivial. Simply set $w_n^j$ to the weight accumulated while running $\mathbf{t}$ in step (1), or $r_{\mathbf{t},n-1}(\hat{s}_{n-1}^j)$ in step (5). The empirical distribution given by $\{(s_n^j, w_n^j)\}_{j=1}^J$ approximates $\langle\!\langle \mathbf{t} \rangle\!\rangle_n / Z_{\langle\!\langle \mathbf{t} \rangle\!\rangle_n}$.
3. **Termination:** If all samples $r_{\mathbf{t}}(s_n^j)$ are values, terminate and output $\{(s_n^j, w_n^j)\}_{j=1}^J$. If not, go to the next step.
   We cannot evaluate values further, so running the algorithm further if all samples are values is pointless. When terminating, assuming the conditions in Theorem 1 or Theorem 2 holds, $\{(s_n^j, w_n^j)\}_{j=1}^J$ approximates $\langle\!\langle \mathbf{t} \rangle\!\rangle / Z_{\langle\!\langle \mathbf{t} \rangle\!\rangle_n}$. Also, by the definition of $[\![ \mathbf{t} ]\!]$, $\{(r_{\mathbf{t}}(s_n^j), w_n^j)\}_{j=1}^J$ approximates $[\![ \mathbf{t} ]\!] / Z_{[\![ \mathbf{t} ]\!]_n}$, the normalized version of $[\![ \mathbf{t} ]\!]$.
4. **Selection:** Resample the empirical distribution $\{(s_n^j, w_n^j)\}_{j=1}^J$. The new empirical distribution is unweighted and given by $\{\hat{s}_n^j\}_{j=1}^J$. This distribution also approximates $\langle\!\langle \mathbf{t} \rangle\!\rangle_n / Z_{\langle\!\langle \mathbf{t} \rangle\!\rangle_n}$.
5. **Mutation:** Increment $n$. Draw $s_n^j \sim k_{\mathbf{t},n}(\hat{s}_{n-1}^j, \cdot)$ for $1 \leq j \leq J$.
   That is, simply run the intermediate program $r_{\mathbf{t},n-1}(\hat{s}_{n-1}^j)$, and draw from $\mathcal{U}(0,1)$ whenever required by a $\texttt{sample}_D$. Record these draws and append them to $\hat{s}_{n-1}^j$, resulting in the trace $s_n^j$. Stop when reaching a term of the form $\mathbf{E}[\texttt{resample}]$ or a value $\mathbf{v}$. The empirical distribution $\{s_n^j\}_{j=1}^J$ approximates $\langle \mathbf{t} \rangle_n / Z_{\langle \mathbf{t} \rangle_n}$. Go to (2).

**Lemma 10.** *Let $n > 0$. If $f_{\mathbf{t},n}(s) > 0$, then $f_{\mathbf{t},n}(s) = f_{\mathbf{t},n-1}(\underline{s}) f_{r_{\mathbf{t},n-1}(\underline{s}),1}(\overline{s})$ for exactly one decomposition $\underline{s} * \overline{s} = s$. If $f_{\mathbf{t},n}(s) = 0$, then $f_{\mathbf{t},n-1}(\underline{s}) f_{r_{\mathbf{t},n-1}(\underline{s}),1}(\overline{s}) = 0$ for all decompositions $\underline{s} * \overline{s} = s$. As a consequence, if $f_{\mathbf{t},n}(s) > 0$, then $p_{r_{\mathbf{t},n-1}(\underline{s}),1}(\overline{s}) = 1$.*†

This gives a more efficiently computable definition of the density.

**Lemma 11.** *For $n \in \mathbb{N}$, $\langle \mathbf{t} \rangle_n(S) = \int_S f_{\mathbf{t},n-1}(\underline{s}) p_{r_{\mathbf{t},n-1}(\underline{s}),1}(\overline{s}) d\mu_{\mathbb{S}}(s)$, where $\underline{s} * \overline{s} = s$ is the unique decomposition from Lemma 10.*†[8]

Since the kernels $k_{\mathbf{t},n}$ are sub-probability kernels, the measures $\langle \mathbf{t} \rangle_n$ are finite given that the $\langle\!\langle \mathbf{t} \rangle\!\rangle_n$ are finite.

**Lemma 12.** *$\langle \mathbf{t} \rangle_0$ is a sub-probability measure. Also, if $\langle\!\langle \mathbf{t} \rangle\!\rangle_{n-1}$ is finite, then $\langle \mathbf{t} \rangle_n$ is finite.*†

As discussed in Section 6.2, the $\langle\!\langle \mathbf{t} \rangle\!\rangle_n$ are finite, either by assumption (Theorem 1) or as a consequence of the dominating function of Theorem 2. From this

[8] We only give a proof sketch for this lemma.

and Lemma 12, the $\langle \mathbf{t} \rangle_n$ are also finite. Furthermore, checking that $\langle \mathbf{t} \rangle_n$ are valid, i.e. that the density $f_{\langle \mathbf{t} \rangle_n}$ of each $\langle \mathbf{t} \rangle_n$ covers the density $f_{\langle\!\langle \mathbf{t} \rangle\!\rangle_n}$ of $\langle\!\langle \mathbf{t} \rangle\!\rangle_n$ is trivial. As such, by Lemma 5, we can now correctly approximate $\langle\!\langle \mathbf{t} \rangle\!\rangle_n$ using Algorithm 1. The details are given in Algorithm 2, which closely resembles the standard SMC algorithm in WebPPL. For ease of notation, we assume it possible to draw samples from $\langle \mathbf{t} \rangle_0$ and $k_{\mathbf{t},n}(s, \cdot)$, even though these are sub-probability measures. This essentially corresponds to assuming evaluation never gets stuck or diverges. Making sure this is the case is not within the scope of this paper. The weights in Algorithm 2 at time step $n$ can easily be calculated according to the following lemma.

**Lemma 13.** $w_n(s) = \dfrac{f_{\langle\!\langle \mathbf{t} \rangle\!\rangle_n}(s)}{f_{\langle \mathbf{t} \rangle_n}(s)} = \begin{cases} f_{r_{\mathbf{t},n-1}(\underline{s}),1}(\overline{s}) & \textit{if } n > 0 \\ f_{\mathbf{t},0}(s) & \textit{if } n = 0 \end{cases}$ *when* $f_{\langle \mathbf{t} \rangle_n}(s) > 0$. *Here,* $\underline{s} * \overline{s} = s$ *is the unique decomposition from Lemma 10.*†

#### 7.2 Other SMC Algorithms

In this section, we discuss SMC algorithms other than the BPF.

First, we have the *resample-move* algorithm by Gilks and Berzuini [11], which is also implemented in WebPPL [13], and treated by Chopin [6] and Ścibior et al. [33]. In this algorithm, the SMC kernel is composed with a suitable MCMC kernel, such that one or more MCMC steps are taken for each sample after each resampling. This helps with the so-called degeneracy problem in SMC, which refers to the tendency of SMC samples to share a common ancestry as a result of resampling. We can directly achieve this algorithm in our context by simply choosing appropriate transition kernels in Algorithm 1. Let $k_{\mathrm{MCMC},n}$ be MCMC transition kernels with $\widetilde{\pi}_{n-1} = \langle\!\langle \mathbf{t} \rangle\!\rangle_{n-1}$ as *invariant distributions*. Using the bootstrap kernels as the main kernels, we let $k_n = k_{\mathbf{t},n} \circ k_{\mathrm{MCMC},n}$ where $\circ$ denotes kernel composition. The sequence $k_n$ is valid because of the validity of the main SMC kernels and the invariance of the MCMC kernels.

While Algorithm 1 captures different SMC algorithms by allowing the use of different kernels, some algorithms require changes to Algorithm 1 itself. The first such variation of Algorithm 1 is the *alive* particle filter, recently discussed by Kudlicka et al. [19], which reduces the tendency to degeneracy by not including sample traces with zero weight in resampling. This is done by repeating the selection and mutation steps (for each sample individually) until a trace with non-zero weight is proposed; the corresponding modifications to Algorithm 1 are straightforward. The unbiasedness result of Kudlicka et al. [19] can easily be extended to our PPL context, with another minor modification to Algorithm 1.

Another variation of Algorithm 1 is the auxiliary particle filter [28]. Informally, this algorithm allows the selection and mutation steps of Algorithm 1 to be guided by future information regarding the weights $w_n$. For many models, this is possible since the weighting functions $w_n$ from Algorithm 1 are often parametric in an explicitly available sequence of *observation data points*, which can also be used to derive better kernels $k_n$. Clearly, such optimizations are model-specific, and can not directly be applied in expressive PPL calculi such as ours. However, the general idea of using look-ahead in general-purpose PPLs to guide selection and mutation is interesting, and should be explored.

### 8 Related Work

The only major previous work related to formal SMC correctness in PPLs is Ścibior et al. [33] (see Section 1). They validate both the BPF and the resample-move SMC algorithms in a denotational setting. In a companion paper, Ścibior et al. [32] also give a Haskell implementation of these inference techniques.

Although formal correctness proofs of SMC in PPLs are sparse, there are many languages that implement SMC algorithms. Goodman and Stuhlmüller [14] describe SMC for the probabilistic programming language WebPPL. They implement a basic BPF very similar to Algorithm 2, but do not show correctness with respect to any language semantics. Also, related to WebPPL, Stuhlmüller et al. [36] discuss a coarse-to-fine SMC inference technique for probabilistic programs with independent sample statements.

Wood et al. [43] describe PMCMC, an MCMC inference technique that uses SMC internally, for the probabilistic programming language Anglican [37]. Similarly to WebPPL, Anglican also includes a basic BPF similar to Algorithm 2, with the exception that every execution needs to encounter the same number of calls to `resample`. They use various types of empirical tests to validate correctness, in contrast to the formal proof found in this paper. Related to Anglican, a brief discussion on resample placement requirements can be found in van de Meent et al. [41].

Birch [25] is an imperative object-oriented PPL, with a particular focus on SMC. It supports a number of SMC algorithms, including the BPF [16] and the auxiliary particle filter [28]. Furthermore, they support dynamic analytical optimizations, for instance using locally-optimal proposals and Rao–Blackwellization [24]. As with WebPPL and Anglican, the focus is on performance and efficiency, and not on formal correctness.

There are quite a few papers studying the correctness of MCMC algorithms for PPLs. Using the same underlying framework as for their SMC correctness proof, Ścibior et al. [33] also validates a trace MCMC algorithm. Another proof of correctness for trace MCMC is given in Borgström et al. [3], which instead uses an untyped lambda calculus and an operational semantics. Much of the formalization in this paper is based on constructions used as part of their paper. For instance, the functions $f_{\mathbf{t}}$ and $r_{\mathbf{t}}$ are defined similarly, as well as the measure space $(\mathbb{S}, \mathcal{S}, \mu_{\mathbb{S}})$ and the measurable space $(\mathbb{T}, \mathcal{T})$. Our measurability proofs of $f_{\mathbf{t}}$, $r_{\mathbf{t}}$, $f_{\mathbf{t},n}$, and $r_{\mathbf{t},n}$ largely follow the same strategies as found in their paper. Similarly to us, they also relate their proof of correctness to classical results from the MCMC literature. A difference is that we use inverse transform sampling, whereas they use probability density functions. As a result of this, our traces consist of numbers on $[0, 1]$, while their traces consist of numbers on $\mathbb{R}$. Also, inverse transform sampling naturally allows for built-in discrete distributions. In contrast, discrete distributions must be encoded in the language itself when using probability densities. Another difference is that they restrict the arguments to `weight` to $[0, 1]$, in order to ensure the finiteness of the target measure.

Other work related to ours include Jacobs [17], Vákár et al. [39], and Staton et al. [35]. Jacobs [17] discusses problems with models in which `observe` (related to `weight`) statements occur conditionally. While our results show that SMC inference for such models is correct, the models themselves may not be useful. Vákár et al. [39] develops a powerful domain theory for term recursion in PPLs, but does not cover SMC inference in particular. Staton et al. [35] develops both operational and denotational semantics for a PPL calculus with higher-order functions, but without recursion. They also briefly mention SMC as a program transformation.

Classical work on SMC includes Chopin [6], which we use as a basis for our formalization. In particular, Chopin [6] provides a general formulation of SMC, placing few requirements on the underlying model. The book by Del Moral [7] contains a vast number of classical SMC results, including the law of large numbers and unbiasedness result from Lemma 5. A more accessible summary of the important SMC convergence results from Del Moral [7] can be found in Naesseth et al. [26].

### 9 Conclusions

In conclusion, we have formalized SMC inference for an expressive functional PPL calculus, based on the formalization by Chopin [6]. We showed that in this context, SMC is correct in that it approximates the target measures encoded by programs in the calculus under mild conditions. Furthermore, we illustrated a particular instance of SMC for our calculus, the bootstrap particle filter, and discussed other variations of SMC and their relation to our calculus.

As indicated in Section 2, the approach used for selecting resampling locations can have a large impact on SMC accuracy and performance. This leads us to the following general question: can we select optimal resampling locations in a given program, according to some formally defined measure of optimality? We leave this important research direction for future work.

**Acknowledgments**

We thank our colleagues Lawrence Murray and Fredrik Ronquist for fruitful discussions and ideas. We also thank Sam Staton and the anonymous reviewers at ESOP for their detailed and helpful comments.
